# Staphylococcal Peptidoglycan Co-Localizes with Nod2 and TLR2 and Activates Innate Immune Response via Both Receptors in Primary Murine Keratinocytes

**DOI:** 10.1371/journal.pone.0013153

**Published:** 2010-10-07

**Authors:** Maria Anna Müller-Anstett, Patrick Müller, Till Albrecht, Mulugeta Nega, Jeanette Wagener, Qiang Gao, Susanne Kaesler, Martin Schaller, Tilo Biedermann, Friedrich Götz

**Affiliations:** 1 Microbial Genetics, University of Tübingen, Tübingen, Germany; 2 Department of Dermatology, University of Tübingen, Tübingen, Germany; New York University, United States of America

## Abstract

In mammalian host cells staphylococcal peptidoglycan (PGN) is recognized by Nod2. Whether PGN is also recognized by TLR2 is disputed. Here we carried out PGN co-localization and stimulation studies with TLR2 and Nod2 in wild type and mutant host cells. To exclude contamination with lipoproteins, polymeric staphylococcal PGN (PGN_pol_) was isolated from *Staphylococcus aureus Δlgt* (lacking lipidated prelipoproteins). PGN_pol_ was biotinylated (PGN-Bio) for fluorescence monitoring with specific antibodies. Keratinocytes from murine oral epithelium (MK) readily internalized PGN-Bio in an endocytosis-like process. In wt MK, PGN_pol_ induced intracellular accumulation of Nod2 and TLR2 and co-localized with Nod2 and TLR2, but not with TLR4. In TLR2-deficient MK Nod2 and in Nod2-deficient MK TLR2 was induced, indicating that PGN_pol_ recognition by Nod2 is independent of TLR2 and vice versa. In both mutants IL-6 and IL-1B release was decreased by approximately 50% compared to wt MK, suggesting that the immune responses induced by Nod2 and TLR2 are comparable and that the two receptors act additively in MK. In TLR2-tranfected HEK293 cells PGN_pol_ induced NFkB-promoter fused luciferase expression. To support the data, co-localization and signaling studies were carried out with SHL-PGN, a lipase protein covalently tethered to PGN-fragments of varying sizes at its C-terminus. SHL-PGN also co-localized with Nod2 or TLR2 and induced their accumulation, while SHL without PGN did not. The results show that staphylococcal PGN not only co-localizes with Nod2 but also with TLR2. PGN is able to stimulate the immune system via both receptors.

## Introduction

*S. aureus* is one of the most clinically important inflammation-inducing Gram positive pathogens. Under these circumstances it is surprising how contradictory results concerning host immune stimulation are. Some of these conflicting results are due to contaminations in the macromolecules used to study microbial associated molecular pattern (MAMP) activity. The important role of lipoproteins became obvious by comparative analysis of Δ*lgt* mutants, which were affected in lipidation of pro-lipoproteins, with wt *S. aureus*; The immune stimulation of different host cells was drastically decreased in these mutants [1]. It emerged over time that, in Gram-positive bacteria, lipoproteins and not lipoteichoic acid (LTA) play a key role in stimulation of the innate immunity [1], [2], [3], [4]. The role of lipoproteins was also studied *in vivo*. In a sepsis model with C57BL/6 mice, the Δ*lgt* mutants of *S. aureus* SA113 and Newman induced much less IL-1B chemokine-mediated inflammation and were virulence attenuated mainly because of their impaired iron acquisition [5]. *In vitro* assays and co-crystallization studies show that Lpp are TLR2 ligands and stimulate the immune system via TLR2 [6], [7], [8]. TLR1 and TLR6, which can form TLR2 heterodimers, are not necessary for immune system activation [9], [10]. The important role of TLR2 and the major adapter protein MyD88 in signaling Lpp was also demonstrated in corresponding knockout mice [5]. In addition to Lpp, TLR2 is also described to recognize lipo-arabinomannan and porins from *Neisseria* [11].

While the role of Lpp in TLR2 signaling is widely accepted, there are some conflicting results regarding the host receptors for peptidoglycan (PGN). Staphylococci and streptococci have lysine (Lys)-type PGN. In *S. aureus* PGN is modified by O-acetylation at the C6-OH position of MurNAc, which contributes to lysozyme resistance [12]. Whether O-acetylation affects signaling activity has not been systematically investigated. However, it has been shown that O-acetylation of PGN strongly suppresses phagocytosis, inflammasome activation, and IL-1beta secretion [13]. PGN fragments are released into the culture supernatant by the natural cell wall turnover [14], [15] and it is therefore only logical that PGN, as a unique bacterial structure, is recognized by the immune system. In mammals there are two intracellular receptors for PGN, Nod2 detects (Lys)-type PGN and muramyl dipeptide (MDP) as the minimal recognition structure, whereas Nod1 preferentially senses the diaminopimelate-containing GlcNAc-MurNAc tripeptide muropeptide found mostly in Gram-negative PGN [16], [17], [18]. PGN can be also sensed by so called PGN-recognition proteins (PGRPs) present in leucocytes, liver and epithelial cells [19], [20], [21], [22].

While the role of Nod2 as a receptor for Gram-positive PGN is well documented, reports as to the role of TLR2 as a PGN receptor are contradictory. In some studies TLR2 is described as a receptor for PGN [23], [24], [25], [26], [27], [28], [29]. Other reports show that both MDP and highly purified PGN from several bacteria was not detected by TLR2 [30], [31]. In the scientific community there is still a tendency to believe that TLR2 is not stimulated by PGN.

To resolve some of the questions regarding PGN stimulation *via* TLR2 we carried out co-localization studies with polymeric PGN (PGN_pol_) and Nod2, TLR2 and TLR4 in various host cells. The aim of the study was to unambiguously prove or disprove that TLR2 is a receptor for PGN and that PGN stimulates the immune system in a TLR2 dependent manner. Due to the known pitfalls with the purification of macromolecules we took great care in the isolation and purification of PGN, and we used two different PGN sources, naked polymeric PGN and protein coupled PGN (SHL-PGN). Co-localization and signaling studies were predominantly carried out with keratinocytes from murine oral epithelium (MK). Our data indicate that staphylococcal PGN is a TLR2 ligand.

## Results

### Biotinylation as a method for fluorescence detection of PGN_pol_

**Figure 1:**
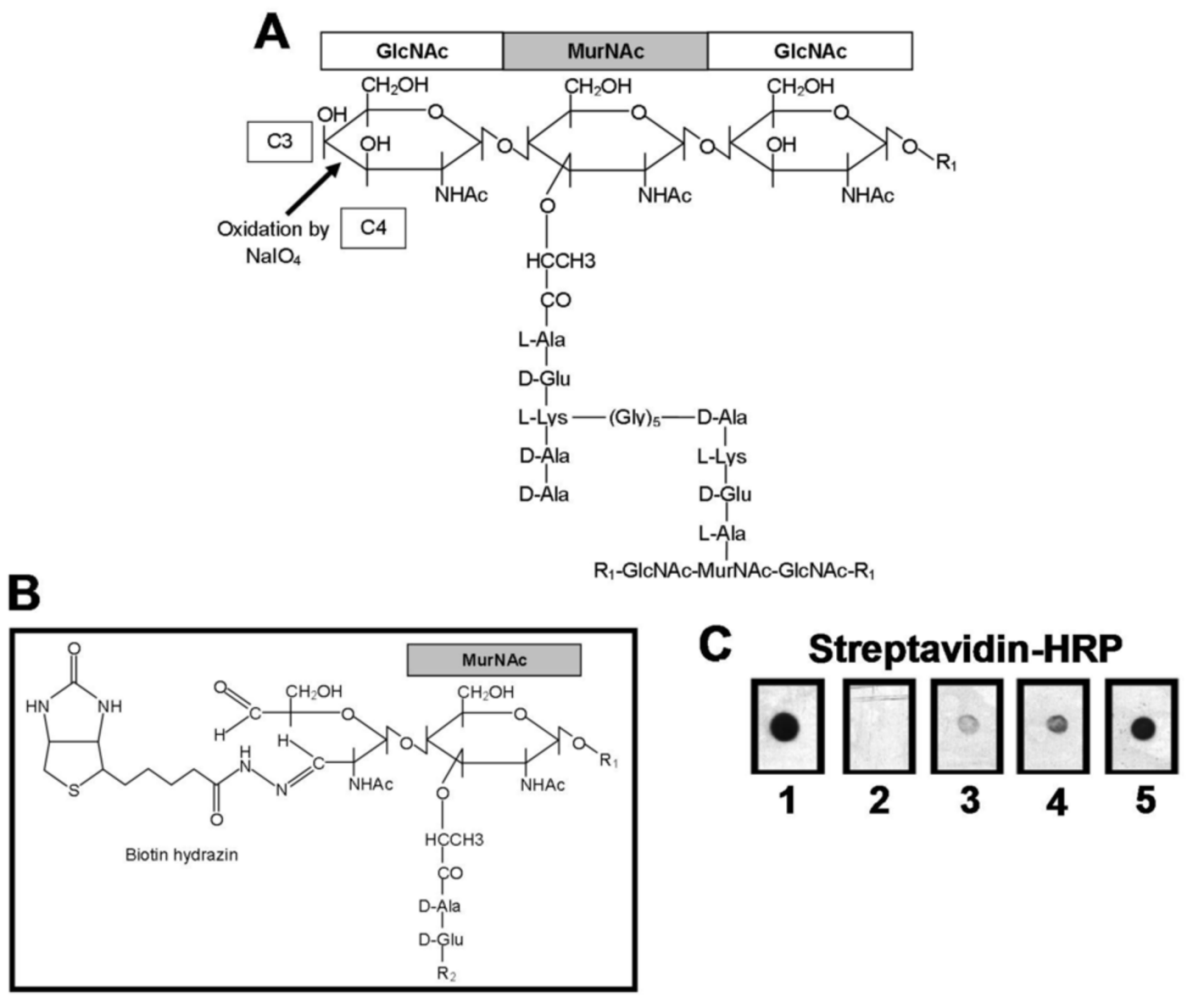
Oxidation and biotinylation of PGN_pol_ from *S. aureus* SA113 Δ*lgt*. (**A**) PGN from *S. aureus* SA113 Δ*lgt* lacks lipoproteins. Covalently coupled proteins and non-covalently associated proteins, WTA, LTA, and O-acetylation were removed from PGN during purification. Oxidation by NaIO_4_ only occurs between C3 and C4 of the terminal GlcNAc at the non-reducing end of the glycan strand. (**B**) The oxidation reaction cleaved the GlcNAc ring and led to two free aldehyde groups, which were used for biotinylation with biotin-hydrazide. In the figure, one biotin-hydrazide reacted with one of the two aldehyde groups. The remaining free aldehyde group may also react with a biotin-hydrazide. (**C**) Verification of biotinylation by dot blot analysis. Biotin (1), non-oxidized PGN_pol_ incubated with 10 µg of biotin-hydrazide (2) 1, 2 and 10 µg of PGN-Bio (3–5) were dropped onto a nitrocellulose membrane. Biotin was detected via streptavidin-HRP and visualized by ECL. Only the oxidized PGN_pol_ was biotinylated. PGN without free aldehyde groups remained unlabeled. GlcNAc: N-acetyl glucosamine; MurNAc: N-acetyl muramic acid; R_1_: [GlcNAc-MurNAc]_n_; R_2_: L-Lys-D-Ala-D-Ala linked by [Gyl]_5_ to another PGN strand.

The question whether the observed stimulatory effect of PGN towards TLR2 is due to contaminations is still a controversial one [31]. Therefore, we adopted a very stringent purification protocol for PGN_pol_ to obtain high purity. To avoid contaminations with lipoproteins, which are strong immune modulators [1], [3], PGN_pol_ was isolated from SA113 Δ*lgt*, which is defective in lipidation of prelipoproteins. Purified PGN_pol_ was also verified by HPLC analysis after mutanolysin digestion (**Fig S1**). As purified PGN_pol_ hardly contains free reactive groups, labeling with most commercial available fluorescent dyes failed (data not shown). Therefore, we established a method to biotinylate purified PGN_pol_ for visualization. The oxidizing reagent sodium-*meta*-periodate (NaIO_4_) was used to cleave the chemical bond between the C3- and C4-atom of the terminal GlcNAc at the non-reducing end of the glycan strand [32]. This selective reaction led to the formation of two free aldehyde groups at the non-reducing end of the glycan strands (**Fig 1A and B**). The biotinylation of the oxidized PGN_pol_ was verified by dot blot analysis (**Fig 1C**).

**Figure 2:**
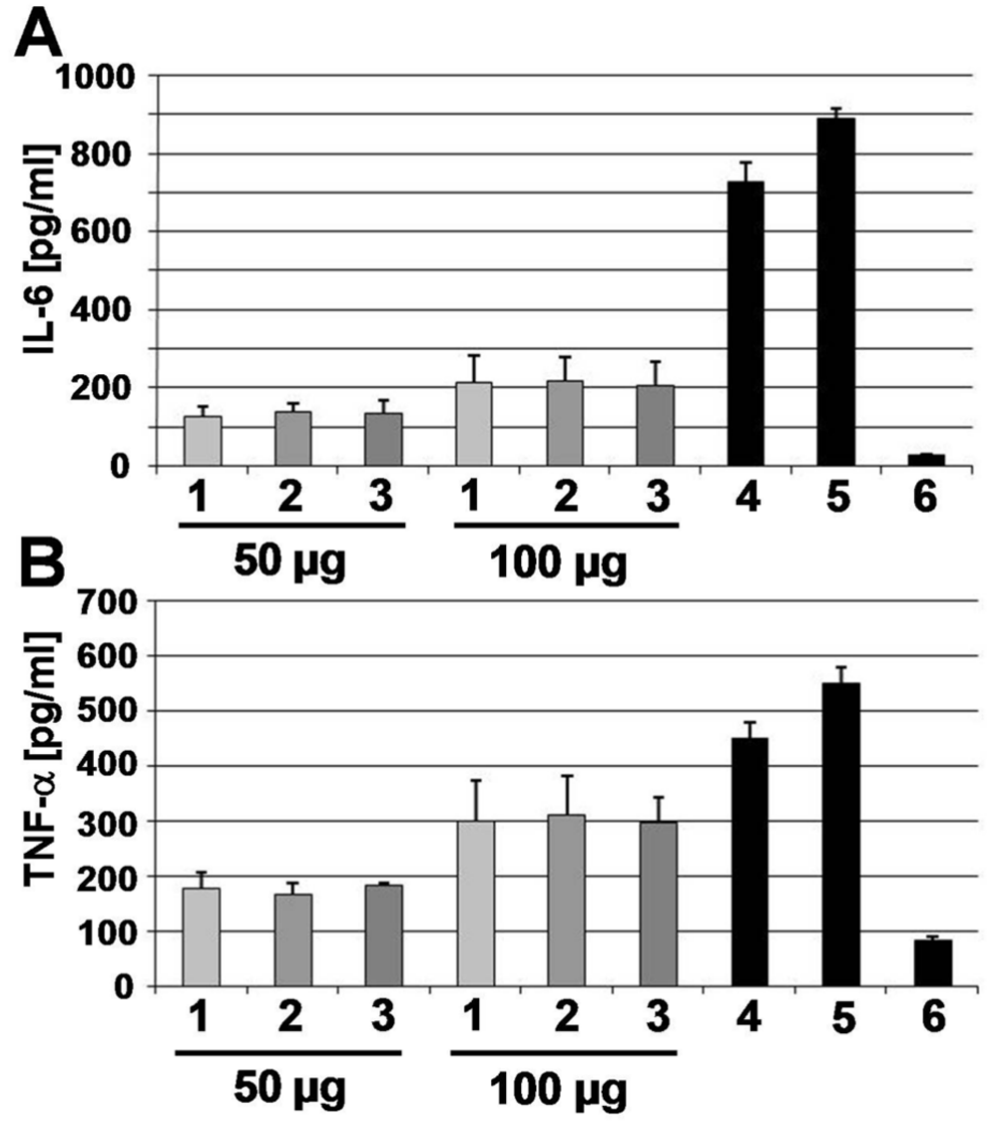
Release of IL-6 and TNF-α after stimulation with PGN-Bio. MonoMac 6 were stimulated with different amounts of untreated PGN_pol_ (1), oxidized PGN_pol_ (2), biotinylated PGN_pol_ from *S. aureus* 113 Δ *lgt* (PGN-Bio) (3), 1 µg/ml Pam_3_Cys (4) and 1 µg/ml LPS (5). (6): Non-stimulated MonoMac 6. Supernatant was analyzed by ELISA. Untreated PGN_pol_, oxidized PGN_pol_, and PGN-Bio induced the release of TNF-α after 4h (**A**) and the release of IL-6 after 24 h of stimulation (**B**), respectively. No significant change in the signaling activity of PGN_pol_ after oxidation/biotinylation was detectable.

As the average length of the glycan strands in *S. aureus* is ∼10 disaccharides and non-reducing ends are terminated either by GlcNAc or MurNAc, only about 5% of all GlcNAc within the PGN network will be oxidized. However, the oxidation of even a low percentage of GlcNAc within PGN may change the signaling activity towards PGN-recognizing PRRs. To test this, human monocytes (MonoMac 6) were stimulated with different amounts of untreated PGN_pol_, oxidized PGN_pol_ and biotinylated PGN_pol_ (PGN-Bio). The amount of TNF-α and IL-6 in the culture supernatants was determined by ELISA. As PGN_pol_ biotinylation (PGN-Bio) had no effect on the release of TNF-α or IL-6 (**Fig 2A and 2B**), PGN-Bio was further used for co-localization studies with host PRRs in primary host cells.

### PGN-Bio elicited intracellular accumulation of Nod2 and TLR2 and co-localizes with both PRRs

**Figure 3:**
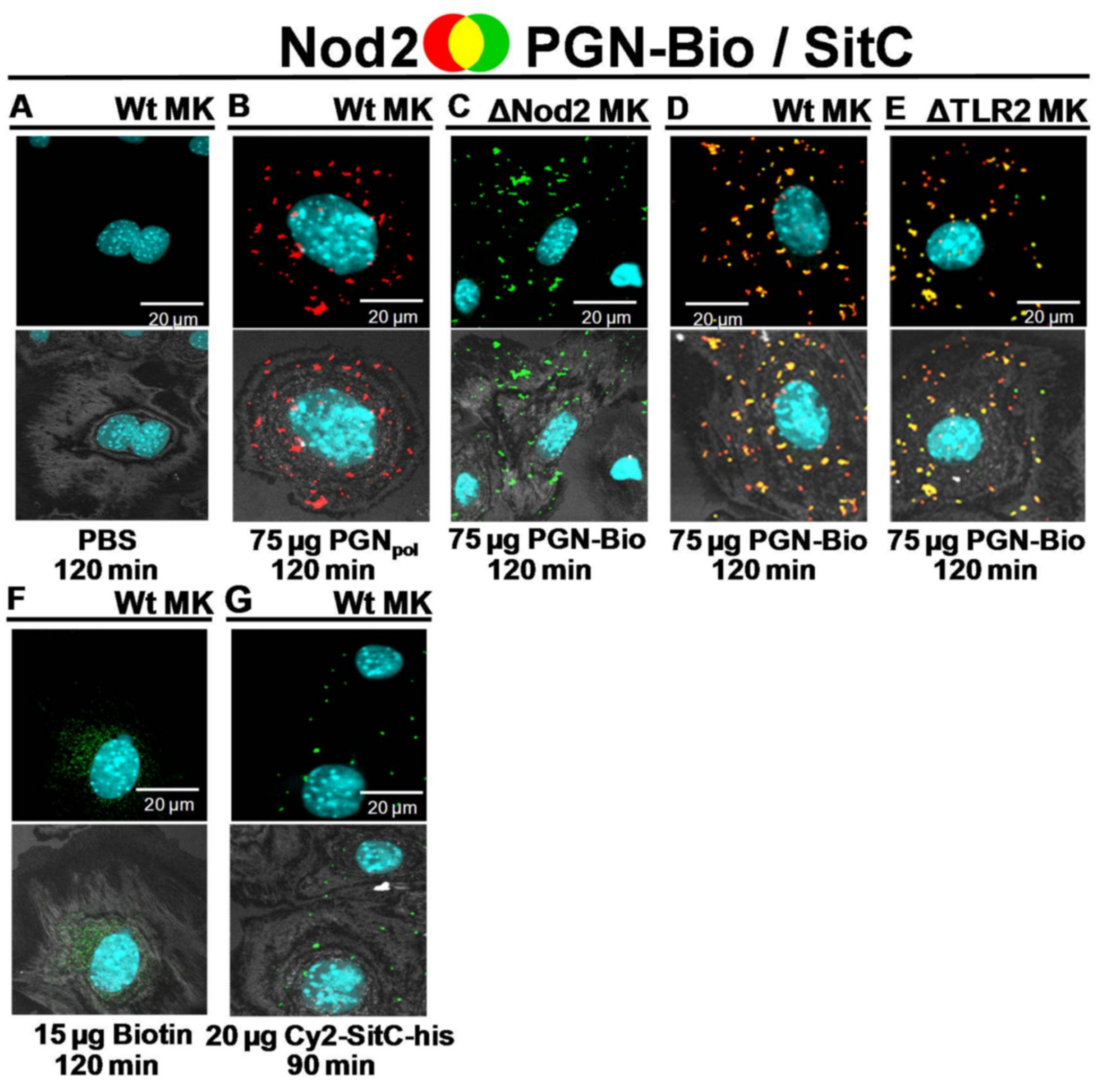
PGN-Bio was incorporated into keratinocytes from murine oral epithelium (MK) and co-localized with Nod2. Confocal images of primary MK stained intracellularly with a rabbit anti-Nod2-antibody (detected by a Cy3-conjugated anti-rabbit antibody [red]). Nuclei were stained with DAPI (blue). PGN-Bio was detected by a FITC-conjugated anti-biotin-antibody (green). The upper panels show the merged images; co-localization events are visualized in yellow. The lower images show an overlay of fluorescence merge and the host cell acquired in reflection mode of the confocal microscope. (**A**) PBS control. (**B**) Stimulation with non-biotinylated PGN_pol_ from *S. aureus* SA113 Δ*lgt* led to Nod2 accumulation. (**C**) PGN-Bio was incorporated into Nod2-deficient MK, but co-localization was not detectable as Nod2 was not present. (**D**) PGN-Bio elicited Nod2 accumulation and co-localized with Nod2. (**E**) In TLR2-deficient MK PGN-Bio was internalized, elicited accumulation of Nod2 and co-localized with Nod2. (**F**) Biotin did not affect Nod2. (**G**) Nod2 was not detected after stimulation with Cy2-SitC-his. Images of cells shown are representative of the cells observed in each dish and are representative of three experiments.

**Figure 4:**
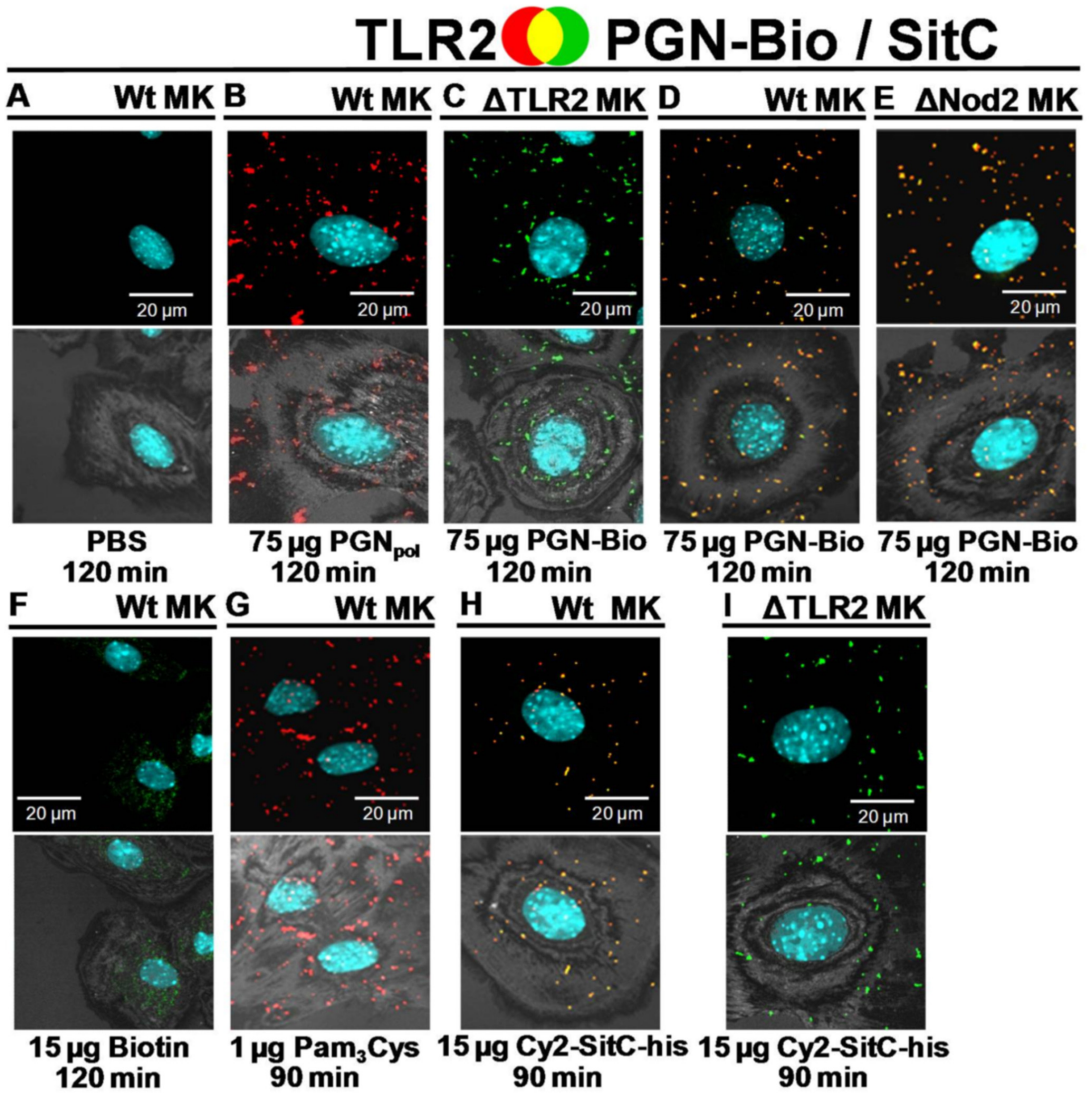
PGN-Bio was incorporated into keratinocytes from murine oral epithelium (MK) and co-localized with TLR2. For a description of the experiment see Fig 3 except for: MK were stained with a rabbit anti-TLR2-antibody and visualized by a Cy3-conjugated anti-rabbit antibody (red). (**A**) PBS control. (**B**) TLR2 was accumulated in wt MK exposed to non-biotinylated PGN_pol_ from *S. aureus* SA113 Δ*lgt*. (**C**) PGN-Bio was incorporated into TLR2-deficient MK. (**D**) Stimulation with PGN-Bio augmented TLR2 occurrence. PGN-Bio and TLR2 showed strong co-localization. (**E**) TLR2 was detected and co-localized with PGN-Bio in Nod2-deficient MK. (**F**) Stimulation with biotin did not affect TLR2. (**G**) The synthetic lipopeptide Pam_3_Cys elicited TLR2 accumulation. (**H+I**) Stimulation with Cy2-SitC-his led to an increased intracellular content of TLR2 in wt MK (H), but not in TLR2-deficient MK (I). Images of cells shown are representative of the cells observed in each dish and are representative of three experiments.

Cell stimulation and subsequent microscopic analysis was carried out in wild type (wt) as well as in Nod2- or TLR2-deficient keratinocytes from murine oral epithelium (MK). In non-stimulated wt MK neither Nod2 (**Fig 3A**) nor TLR2 (**Fig 4A**) were detectable. However, stimulation with either PGN_pol_ or PGN-Bio induced a strong accumulation of both receptors (**Fig 3 B and D**; **Fig 4B and D**). Scanning of 0.4 µm sections revealed that most Nod2 and TLR2 was localized intracellularly. In Nod2-deficient and TLR2-deficient MK, PGN-Bio was incorporated to a similar degree as in wt MK (**Fig 3C** **and** **Fig 4C**), suggesting that the uptake of PGN-Bio in MK is independent of both receptors.

In wt MK, internalized PGN-Bio was detectable after 15 min and Nod2 and TLR2 emerged. Both receptors largely co-localized with PGN-Bio. Significantly higher amounts of PGN-Bio were internalized after 90 min. This correlated with a further increase in Nod2 and TLR2 (**Fig S2**). A maximum was reached after 120 min, as longer stimulation (180 min; data not shown) did not lead to a further increase of intracellular PGN-Bio, Nod2 or TLR2. An increase of PGN-Bio from 75 to 150 µg led to a significant increase of intracellular Nod2. This increase was less pronounced for TLR2 (**Fig S2**).

PGN-Bio induced Nod2 accumulation in TLR2-deficient MK (**Fig 3E**), and TLR2 accumulation in Nod2-deficient MK (**Fig 4E**), indicating, that PGN_pol_ stimulation via Nod2 is independent of TLR2 and *vice versa*. Again, the majority of Nod2 and TLR2 co-localized with PGN-Bio. Biotin was tested as a negative control and showed no effect on Nod2 or TLR2 (**Fig 3F** **and** **4F**). As expected, TLR2 accumulation was also observed after stimulation with the synthetic lipopetide Pam_3_Cys (**Fig 4G**).

**Figure 5:**
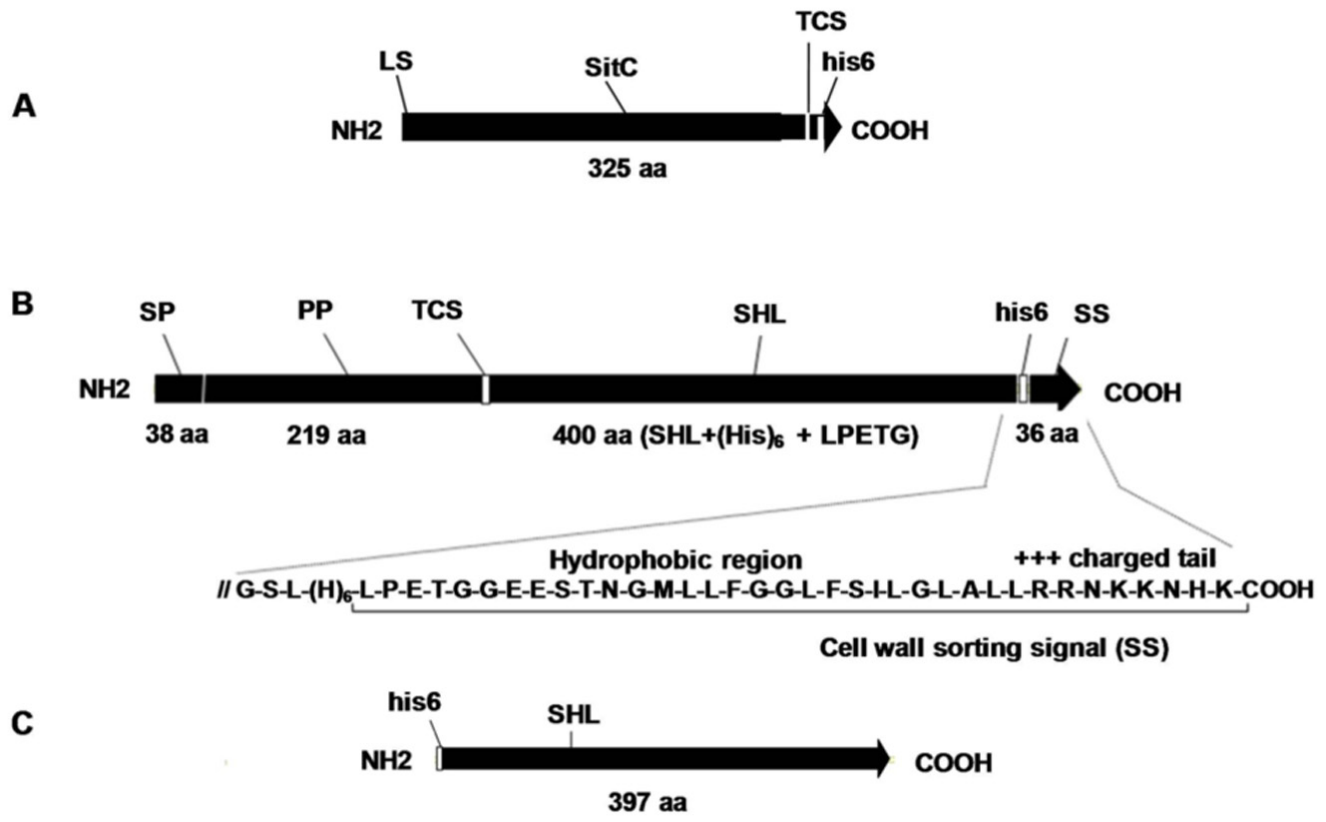
Protein constructs. (**A**) C-terminal histidine-tagged pre-protein of *S. aureus* SitC. (**B**) Cytoplasmically produced SHL histidin-tagged from pPSHG5His6mSHL. (**C**) Sec-translocated and SrtA PGN-anchored histidine-tagged (his6) *S. hyicus* lipase (SHL) hybrid protein. SP: signal peptide, PP: pro-peptide, TCS: thrombin cleavage site, SS: cell wall sorting sequence containing SrtA recognition motif LPETG, hydrophobic and positively charged residues, LS: leader sequence.

**Figure 6:**
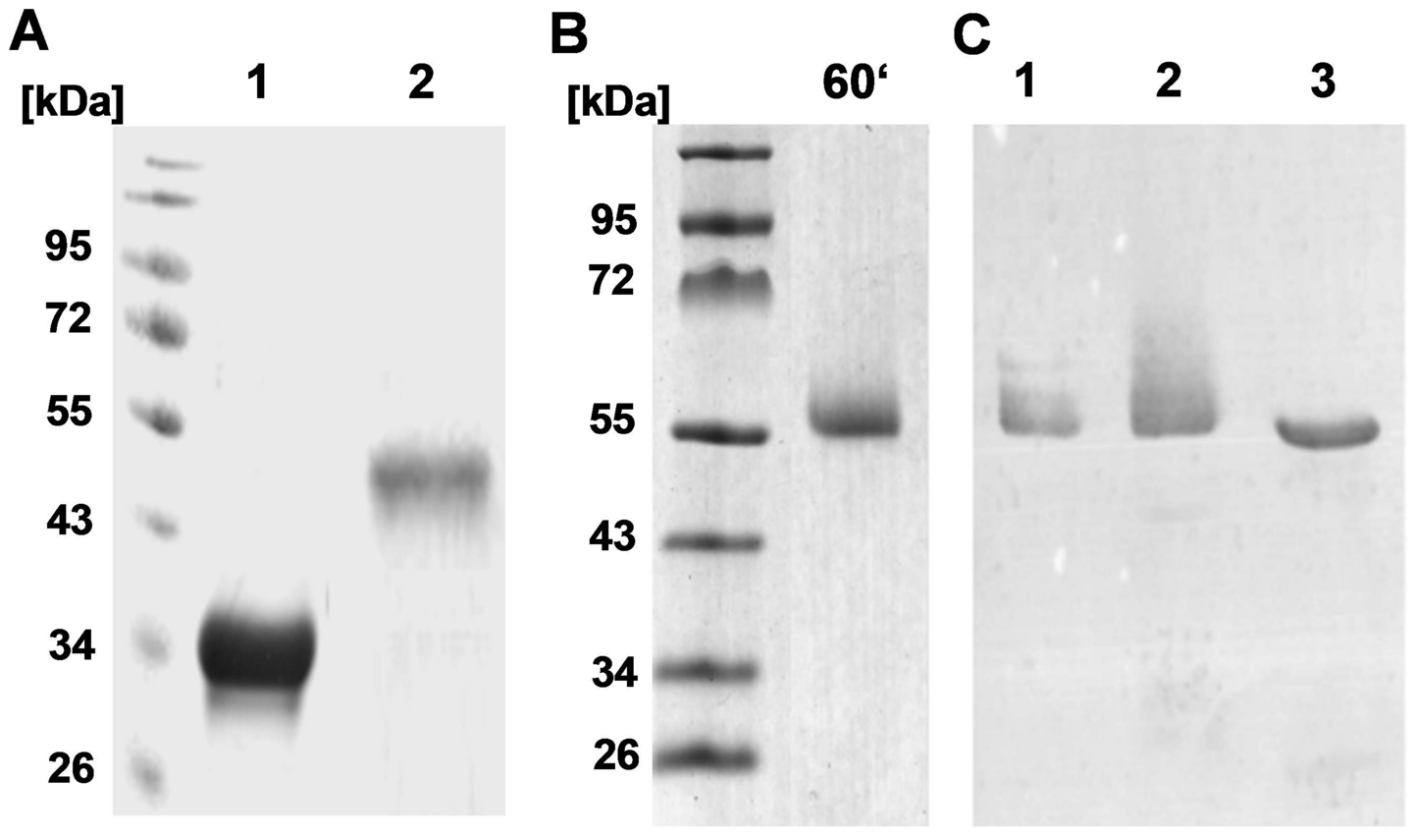
Verification of purified SHL covalently anchored to PGN residues and of purified SHL and SitC-his. (**A**) Coomassie blue stained SDS-PAG of purified SitC-his (lane A1) and SHL (lane A2). (**B**) Coomassie blue stained SDS-PAG of the elution fraction after purification of SHL-PGN from *S. carnosus* (pCX33TLCH). (**C**) Western blot with rabbit α-lipase antibody. Affinity purified SHL-PGN released after 60 min (lane C1) and 120 min (lane C2) of lysozyme treatment. Subsequent treatment of SHL-PGN from lane 2 with lysostaphin for 120 min (lane C3) led to a distinct 52 kDa SHL band devoid of PGN moieties.

The native lipoprotein SitC, one of the most abundant lipoproteins in *S. aureus* [1], was also tested. C-terminally his-tagged SitC (**Fig 5A**) was expressed in *S. carnosus* (pTX30SitC-his), extracted from the cytoplasmic membrane, affinity purified (**Fig 6A**, **lane 1**) and labeled with Cy2 (Cy2-SitC-his). In wt MK, Cy2-SitC-his stimulated TLR2 but not Nod2 accumulation. The majority of TLR2 was co-localized with Cy2-SitC-his (**Fig 3G** **and** **4H**). Cy2-SitC-his showed no stimulatory activity in TLR2-deficient MK (**Fig 4I**), as Lpp are typical TLR2 ligands.

To substantiate the results with PGN_pol_ and PGN-Bio we carried out studies with protein-coupled PGN. A staphylococcal lipase from *S. hyicus* (SHL) covalently anchored to the cell wall of *Staphylococcus carnosus* in its active form was used as a carrier protein [33]. In our construct, SHL was supplied with a his-tag upstream of the sorting sequence (**Fig 5B**). SHL with C-terminally tethered PGN (SHL-PGN) was released from *S. carnosus* (pCX33TLCH) cells by lysozyme treatment, affinity purified (**Fig 6B**) and labeled with Cy5 (Cy5-SHL-PGN). Due to the variable PGN-portion, SHL-PGN appeared as a smear between 55 to 65 kDa with a peak at 60 kDa (**Fig 6B and 6C**, **lanes 1 and 2**). After treatment with lysostaphin, PGN was removed and the SHL appeared as a single band at 52 kDa (**6C**, **lane 3**). As calculated from the mass difference, the PGN moiety consisted of approximately 5–8 PGN monomers. The average mass of *S. aureus* PGN monomer is [M+Na+] 1090.5 *m/z*) [12]. SHL without PGN (**Fig 5C**) was purified (**Fig 6A** **lane 2**) and labeled with Cy5 (Cy5-SHL) as a negative control.

**Figure 7:**
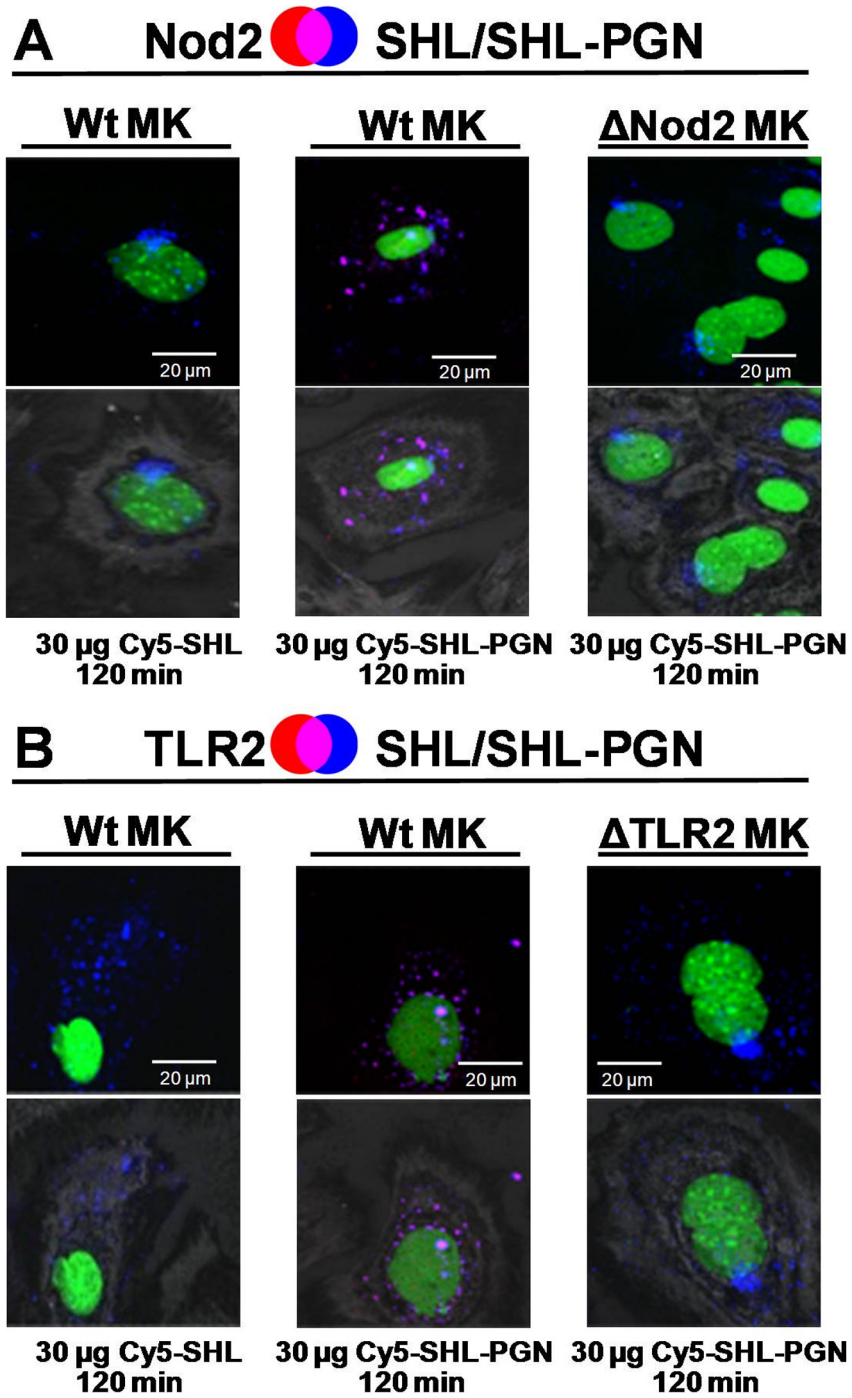
*S. carnosus* TM300 PGN covalently coupled to SHL co-localized with Nod2 and TLR2. Confocal images of MK thin sections stained with anti-Nod2-antibody (**A**) or anti-TLR2-antibody (**B**) detected by a Cy3-conjugated secondary antibody [red]. Nuclei were stained with Yo-Pro (green). SHL-PGN and SHL were directly labeled with Cy5 (blue). The upper panels show the merged fluorescence images; areas of co-localization are shown in pink. The lower images show an overlay of the merged fluorescence images and the host cell acquired in reflection mode of the confocal microscope at 488 nm. Both Cy5-SHL-PGN and Cy5-SHL were internalized into MK. Cy5-SHL-PGN elicited Nod2 and TLR2 accumulation in wt MK, but Cy5-SHL did not. Cy5-SHL-PGN co-localized with both Nod2 and TLR2. In Nod2-deficient MK Nod2 was not detected. In TLR2-deficient MK TLR2 was not detected. However, Cy5-SHL-PGN and Cy5-SHL were internalized in these cells. Images of cells shown are representative of the cells observed in each dish and are representative of three experiments.

Wt, Nod2- and TLR2-deficient MK were stimulated with 30 µg of Cy5-SHL-PGN or Cy5-SHL. Although both Cy5-SHL and Cy5-SHL-PGN were incorporated into MK, only Cy5-SHL-PGN induced TLR2 and/or Nod2 accumulation in wt and corresponding Nod2/TLR2 mutants (**Fig 7**). Furthermore, only Cy5-SHL-PGN co-localized with Nod2 and TLR2. TLR4 was not detected in wt MK after stimulation with PGN-Bio but after stimulation with LPS (**Fig S3**). Stimulation with PGN_pol_, Biotin, Pam_3_Cys, SitC-his, SHL-PGN and SHL did not affect TLR4 in wt cells (data not shown).

### PGN-Bio was internalized in an endocytosis-like process

**Figure 8:**
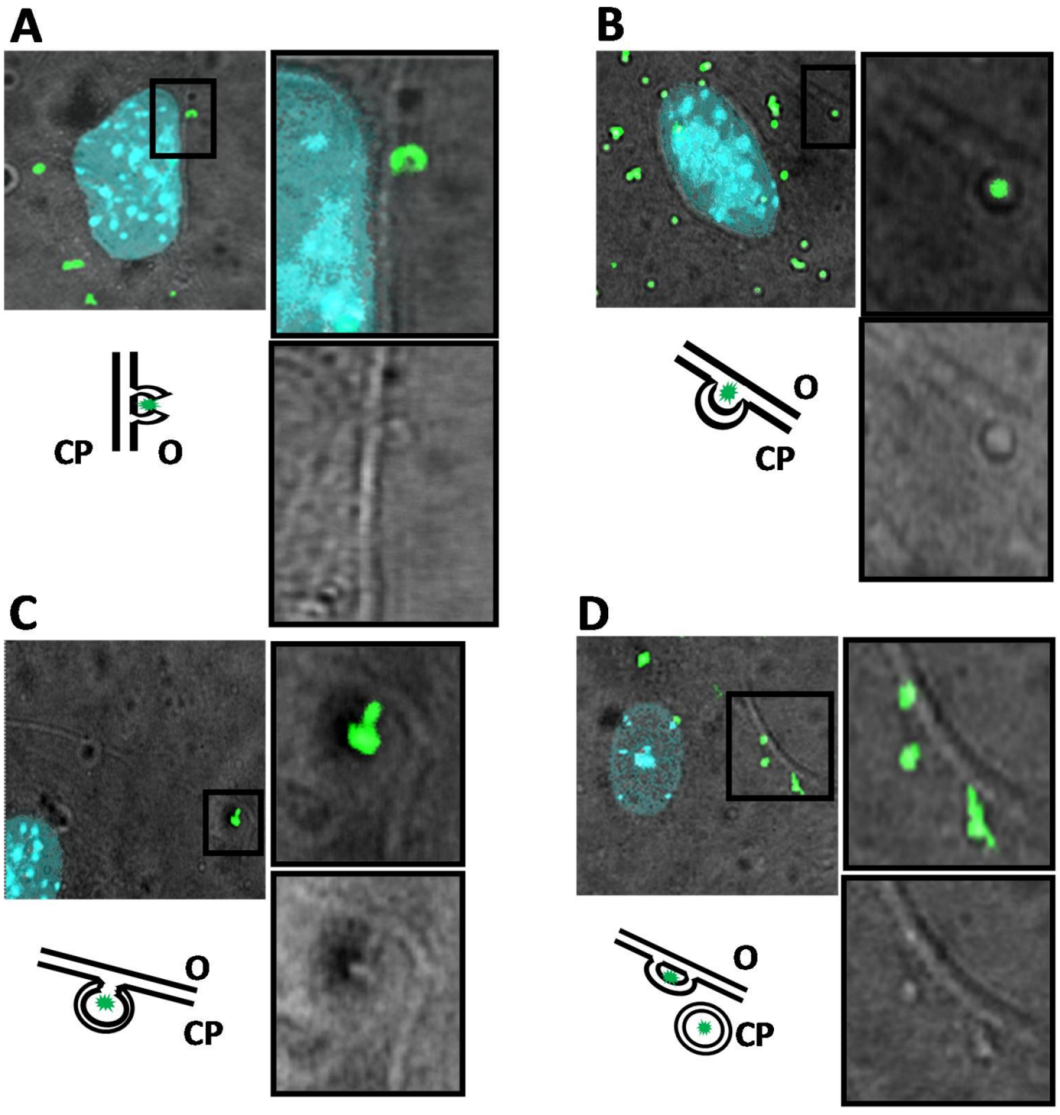
Incorporation of PGN-Bio might occur by endocytosis. Confocal images of keratinocytes from murine oral epithelium (MK). Nuclei were stained with DAPI (blue). PGN-Bio was detected by a FITC-conjugated anti-biotin-antibody (green). The images show an overlay of immuno-detected PGN-Bio and host cell acquired in non-confocal transmitted light channel. (**A**) Membrane protrusion enclosing PGN-Bio. (**B+C**) Internalization of enclosed PGN-Bio. (**D**) Release of the membrane-enclosed PGN-Bio at the inner side of the membrane. O: outside; CP: cytoplasm.

As PGN-Bio is insoluble we raised the question of how PGN_pol_ fragments might reach the cytoplasmic space to finally co-localize with Nod2 inside the cell. A closer look at the adherence of PGN-Bio to the cytoplasmic membrane revealed an engulfment of PGN-Bio by the membrane (**Fig 8A and B**). There are also more advanced steps visible where PGN-Bio is engulfed by host membrane at the inner side of plasma membrane (**Fig 8C**), and where PGN-Bio is finally in the cytoplasm most likely still engulfed in vesicles (**Fig 8D**). This endocytosis-like uptake is Nod2-/TLR2-independent.

### PGN_pol_ (PGN-Bio) induced release of IL-6 and IL-1B and triggered TLR2-specific NFkB-activation

**Figure 9:**
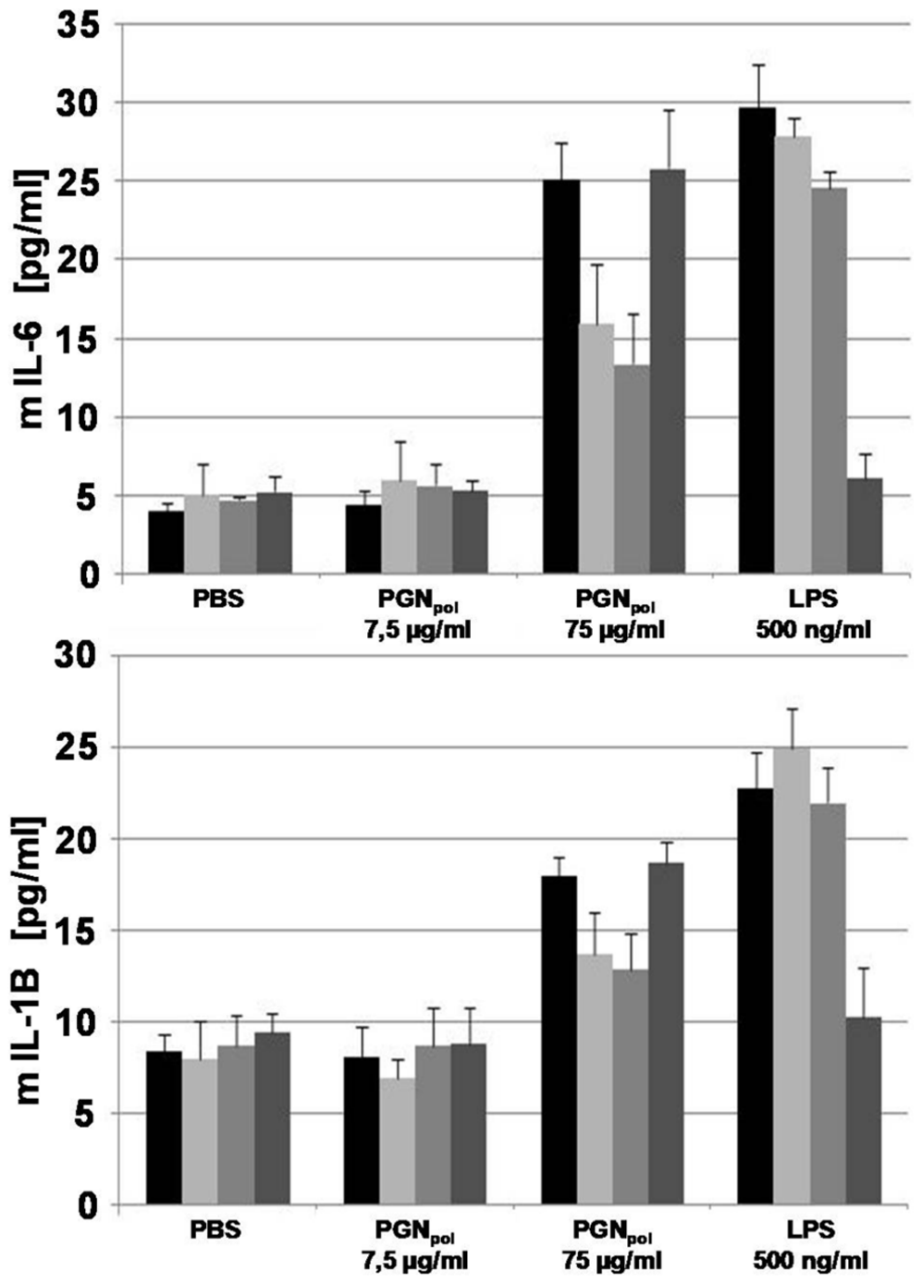
Signaling activity of PGN-Bio in keratinocytes from murine oral epithelium (MK). MK were stimulated with different amounts PGN-Bio and 500 ng/ml LPS for 48 h. The concentrations of murine interleukin 6 and murine interleukin 1B in the culture supernatants were measured using an enzyme-linked immunosorbent assay. Black bars: Wt-MK; light grey bars: TLR2-deficient MK; grey bars: Nod2-deficient MK, dark grey bars: TLR4-deficient MK. The data were shown as the mean +/− S.D. from three independent experiments.

To examine whether the observed co-localization of PGN-Bio with Nod2 and TLR2 finally results in a pro-inflammatory response, the signaling activity of PGN_pol_ in MK was tested. Wt-MK, Nod2-, TLR2- and TLR4-deficient MK were stimulated with PGN_pol_. Cells were treated with PBS as a negative control. IL-6 and IL-1B were produced after stimulation for 48 hours (**Fig 9**). The concentration of IL-6 and IL-1B in the supernatant of Nod2- and TLR2-deficient MK was approximately 50% lower compared to wt-MK and TLR4-deficient MK. This implies that TLR2 and Nod2 contribute equally to the release of IL-6 and IL-1B upon PGN_pol_ stimulation, and furthermore, that TLR2 and Nod2 act additively in wt MK.

We also evaluated the activation of NFκB by PGN_pol_ using HEK293 cells transfected with human TLR2- and Nod2-expressing plasmids. As shown with HEK293/hTLR2 cells, innate immune sensing of PGN_pol_ was clearly TLR2-dependent. PGN_pol_ also caused Nod2 mediated NFkB-activation in HEK293/hNod2 cells (**Fig S4**).

## Discussion

There is mounting evidence that bacterial lipoproteins (Lpp) are TLR2 ligands and induce a strong immune response. However, the role of PGN in innate immunity is not as clear. In most bacteria, PGN fragments are released in varying amounts by the natural cell wall turnover [14], [15]. We also assume that cell wall anchored proteins with covalently bound PGN fragments (e.g. protein A, fibronectin binding proteins, and many more) are released by the same mechanism. For example, Protein A is found in abundance in the culture supernatant of *S. aureus* strains [34], but it has not been verified whether the observed smear in Western blots is due to the varying size of the PGN portion. As PGN is a standard component of all bacterial cell walls, it is not surprising that the immune system is alerted when it appears in the host organism, and it is recognized not only by mammalian but also by plant PRRs [35]. PGN is recognized by Nod1 and Nod2: meso-DAP PGN type (typical for Gram-negative bacteria) is sensed by Nod1 and the lysine PGN type (typical for Gram-positive bacteria) is sensed by the Nod2 receptor [17], [18], [36], [37], [38]. There could also be a connection of Nod2 to Crohn's disease as muramyl dipeptide (MDP) protects mice from experimental colitis by down regulating TLR2 and other TLRs in DCs [39].

As it is widely accepted that Nod2 recognizes PGN, the results whether PGN can be recognized by TLR2 are controversial. Structurally, lipopeptides and PGN are unrelated and the question is whether TLR2 can interact with both. In 1999 it was postulated that PGN activates cells through TLR2 [28]. However, the same paper reported that TLR2 is also activated by LTA, which is, as we now know, unlikely. The upregulation of TLR2 expression in murine primary PMCs by commercial PGN from *S. aureus* was described in several publications [23], [26]. It was also reported that PGN binds to the extracellular domain of recombinant TLR2, suggesting that this domain directly interacts with PGN [40]. This was corroborated recently by binding studies with isolated human TLR2 and synthetic meso-DAP or lysine muropeptides [24]. On the other hand, it has been reported that TLR2 stimulation does not occur via PGN [31]. In contrast, Dziarski and colleagues showed that TLR2 is a PGN receptor using purified PGN form *S. aureus* [25]. The involvement of PGN in TLR2-dependent immune stimulation is still a matter of debate and one must be aware that the observed TLR2-related immunostimulatory activity of PGN could be due to contaminating lipoproteins in all studies in which commercial PGN preparations were used.

Due to the uncertainties and the promiscuous MAMP-recognition of TLR2 [41] we investigated the interaction of purified staphylococcal polymeric PGN (PGN_pol_), which was biotinylated for monitoring reason (PGN-Bio); PGN-Bio showed the same immune stimulating activity as PGN_pol_. As it is so difficult to isolate polymeric PGN that is not contaminated with other potential MAMPs, we isolated and purified PGN_pol_ from the *S. aureus lgt* mutant to rule out lipoprotein contamination. We also tested a second PGN source, where PGN is covalently bound to a lipase as carrier protein (SHL-PGN). Co-localization and signaling studies were carried out with keratinocytes from murine oral epithelium (MK). MKs were chosen, as skin and mucosal keratinocytes are the first responders to external invaders, serve as initiators in innate immunity [42], [43], [44] and express a variety of PRRs [45].

Incubation of PGN-Bio showed that it was readily internalized by both wt, Nod2-, or TLR2-deficient MK, indicating that its uptake is independent of these PRRs. Stimulation with PGN_pol_ or PGN-Bio induced TLR2 and Nod2 production, which accumulated preferentially in the cytoplasm, and reached a maximum after 120 min. In non-stimulated MK neither Nod2 nor TLR2 were detectable, which is in agreement with recent expression studies [46]. Co-localization of PGN Bio with Nod2 and TLR2 was always approximately 80–90%. Signaling studies revealed that PGN_pol_ and PGN-Bio triggered an immune response as measured by the release of murine IL-6 and IL-1B. An interesting question was how Nod2- or TLR2-deficient MK respond upon PGN-Bio stimulation. TLR2-deficient MK induced Nod2, and Nod2-deficient MK induced TLR2, indicating that PGN_pol_ recognition by Nod2 is independent of TLR2 and *vice versa*. However, the amount of IL-6 and IL-1B release was decreased by approximately 50% in the Nod2- or TLR2-MK mutants compared to the wt MK. This suggests that Nod2 and TLR2 have a comparable immune response with PGN_pol_ and act additively in MK. Synergistic activation has been described in human monocytic cells [27]. TLR2-specific immune stimulation by PGN_pol_ was also corroborated with HEK293/hTLR2 cells.

Although our results show that PGN_pol_ stimulates the immune response via TLR2 and Nod2, its stimulating activity appears to be lower compared to Pam_3_Cys or LPS (**Fig 2**). This observation is in agreement with the low stimulating activity of the *lgt* mutants [1], [3].

The construction and isolation of recombinant lipase with covalently tethered PGN was another way to confirm the results with PGN-Bio and PGN_pol_. SHL-PGN had the advantage that it could be purified via Ni-NTA chromatography and the protein portion could be directly Cy5 fluorescence labeled. Furthermore, the affinity purification of SHL-PGN was preceded by 8 M urea treatment, which results in the detachment of noncovalently bound molecules. Another advantage was the availability of SHL without PGN as a control protein, which was purified in a similar way. Co-localization studies with SHL-PGN showed essentially the same results as with PGN-Bio, while Cy5-SHL was unable to induce TLR2 and Nod2 expression, and thus did not show co-localization.

In PGN_pol_-induced MK, the majority of TLR2 was not exposed at the cell membrane but rather intracellularly located. This is consistent with recent findings in human keratinocytes investigated by flow cytometry [46], [47]. It has been shown that TLR2 is recruited to sub-cellular sites, like endolysosomal compartments [48], [49], [50], [51], [52].

This may be because high levels of TLR2 on the cell surface could lead to an overshooting pro-inflammatory cytokine response. Whether intracellular TLR2 can be stimulated is unknown, although it was suggested that intracellularly localized TLR2 may be stimulated after invasion or phagocytosis of pathogens [50]. As *S. aureus* is known to infiltrate various types of host cells, including keratinocytes [53] it would make sense that invasive pathogens activate intracellular TLR2.

SitC is one of the predominant lipoproteins in *S. aureus* [1] and a native TLR2 agonist [54]. In our co-localization studies, we could indeed show that SitC co-localized with TLR2, but not with Nod2. SitC, like Pam_3_Cys induced TLR2 but not Nod2 or TLR4 production. The latter only emerged after stimulation with LPS. PGN-Bio, PGN_pol_, biotin, SHL-PGN, SHL, Pam3Cys, and SitC did not induce TLR4.

The ability of PGN-Bio to activate and co-localize with intracellular Nod2 raises the question of how PGN-Bio penetrates the host membrane. We showed that PGN-Bio was bound to the cell surface and internalized in an endocytosis-like process (**Fig 8**) as has recently been described with HEK293T as a model system [55]. In this model MDP was internalized into host cytosol through endocytosis, most likely by the clathrin-coated pit pathway with the optimal pH for internalization ranging from 5.5 to 6.5.

Finally, it should be mentioned that beside its immune stimulating activity, PGN also affects cell physiology. Recently it has been reported that commercial PGN caused a rapid increase in cytosolic Ca2+, and an increase of phagocytic capacity in mouse DCs [56]. This effect was dependent on voltage-gated K+ (Kv) channel activity. In TLR2-deficient DCs the effect of PGN on [Ca2+]i was dramatically impaired [56]. Unfortunately, commercial PGN was used in this study. Therefore, it is also possible that the observed effect was due to contamination with lipoproteins. However, this study clearly showed that TLR2 interaction with PGN (or Lpp) is accompanied by profound physiological changes.

In conclusion, we demonstrated that TLR2 is a receptor for PGN and that PGN triggers TLR2 specific immune response.

## Materials and Methods

### Bacterial strains, growth media and plasmids

*S. aureus* SA113, *S. aureus* Sa113 Δ*lgt*, *S. carnosus* TM 300, *S. carnosus* (pTX30SitC-his), *S. carnosus* (pCXTLCH), and *S. carnosus* ΔRKET (pPSHGΩHis6mSHL) were grown aerobically at 30 or 37°C in B-medium with the supplements indicated in Table. 1.

**Table 1:** Strains used in this study.

Strain	Relevant Property	Growth Medium Supplement*	Reference
*S. aureus* SA113	laboratory strain	-	[65]
*S. aureus* SA113 Δ*lgt*	deletion of *lgt*	Em	[1]
*S. carnosus* TM300	laboratory strain	-	[66]
*S. carnosus* pTX30SitC-his	induction of *sitC*-his with xylose	Tc	M. Müller-Anstett (this study)
*S. carnosus* pCXTLCH	induction of *shl* (PGN-anchored) with xylose	Cm	T. Albrecht (this study)
*S. carnosus* ΔRKET pPSHG5ΩHis6mSHL	*galRKET*::*aadA* induction of mature SHL with lactose	Cm	Q. Gao (this study)

*) Em, erythromycin; Tc, tetracycline; Cm: chloramphenicol.

### Purification of PGN_pol_

*S. aureus* SA113 Δ*lgt* cells, lacking the diacylglyceryl transferase (lgt), which catalyzes the first step in the biosynthesis of lipoproteins, were grown to an OD_578_ 0.6 and harvested by centrifugation. The pellet was washed with 100 mM Tris/HCl (pH 6.8) and resuspended in the same buffer. A threefold volume of 5% SDS was added and the suspension was dropped into boiling 5% SDS. After 30 min the suspension was allowed to cool down to room temperature (RT). The cell wall was pelleted by ultra centrifugation. The pellet was resuspended in 1 M NaCl. Several washing steps followed to remove SDS. The absence of SDS was determined by Hayashi assay [57]. The cell wall was disrupted with glass beads (FastPrep). Glass beads were removed via a Nutsch filter (Por. 2). Cell wall fragments were spun down by ultra-centrifugation. The pellet was resuspended in buffer A (100 mM Tris/HCl, 20 mM MgSO_4_, pH 7.5) supplemented with DNase and RNase and incubated for 2h at 37°C while stirring. Afterwards CaCl_2_ was added to an end concentration of 20 mM. Covalently bound proteins were removed by incubation of the PGN with 50 µg/ml trypsin for 16 h over night at 37°C. To denature DNase, RNase, and trypsin, SDS was added to a final concentration of 1%. The suspension was incubated for 20 min at 95–100°C. PGN fragments were spun down by ultra-centrifugation and SDS was removed by several washing steps (again determined by Hayashi assay). SDS freed PGN fragments were resuspended in 8 M LiCl and incubated for 15 min at 41°C. LiCl was removed by washing the PGN fragments twice in double deionized water, twice in acetone and twice in double deionized water. PGN fragments were pelleted by ultracentrifugation. The pellet was resuspended in 48% cold HF and stirred 48 h at 4°C. PGN fragments were freed from HF by several alternating washing steps - twice with 50 mM HCl and twice with double deionized water. Finally, the pellet was resuspended in water and lyophilized. The lyophilized highly purified PGN was stored at −20°C.

### Control of PGN_pol_ by High-performance liquid chromatography (HPLC) analysis

Purified peptidoglycan was digested with 100U mutanolysin in 12.5 mM phosphate buffer (pH 5.5) in a total volume of 500µl. Digestions were terminated by incubating the samples at 90°C for 5 min. Insoluble PGN_pol_ was removed by centrifugation, and soluble fractions were dried in a vacuum evaporator. Digested PGN_pol_ was resuspended in water and reduced with sodium borohydride. Excess borohydride was destroyed by adding 20% phosphoric acid. The muropeptide pattern was verified on an Agilent 1200 HPLC with Prontosil 120, 3 µm 250×4.6mm C18 column (Bischoff Chromatography, Leonberg, Germany) using a linear gradient of 100 mM sodium phosphate buffer (pH2.5) to 30% methanol for 150 min. Muropeptides were detected at 205 nm.

### Oxidation and biotinylation of PGN_pol_

Purified PGN_pol_ from *S. aureus* SA113 Δ*lgt* was incubated with 30 mM sodium-meta-periodate (NaIO_4_, Merck, Hohenbrunn, Germany) on ice in the dark for 30 min. To remove the NaIO_4_ the sample was dialyzed (MWCO 8 kDa, 0.1 M sodium acetate, pH 5.5) over night. The oxidized PGN_pol_ was incubated with biotin-hydrazide (Sigma-Aldrich, Taufkirchen, Germany) for 2 h at RT. After another dialyzing step (MWCO 8 kDa, 0.1 M sodium acetate, pH 5.5) that removed non-coupled biotin-hydrazide, LPS concentration was determined by QCL-1000 LAL assay (Table 2). Successful biotinylation of PGN_pol_ was verified by dot blot analysis.

**Table 2:** LPS content in the used preparations.

Stimulant	LPS concentration in 10 µg of preparation
SitC-his	0.005 EU
SHL	0.01 EU
SHL-PGN	0.01 EU
Biotin	0.01 EU
PGN-Bio	0.01 EU
Pam_3_Cys	0.02 EU

LPS concentrations in the stock solutions of the stimulants used were determined by QCL-1000 LAL assay (Cambrex, Walkersville, USA). The given EU values were calculated for 10 µg of each stimulant. 10 EU = 1 ng LPS.

### Purification of SHL-PGN from *S. carnosus* (pCX33TLCH)

The vector pCX30Δ82cw was previously constructed for studies on *in vivo* immobilization of enzymatically active proteins on the staphylococcal cell surface [33]. This vector was used to provide the lipase with a his-tag in front of the 36 aa FnbpB-sorting sequence and a thrombin cleavage site (TCS) between the lipase-propeptide (PP) and the mature lipase. The lipase is covalently anchored to the PGN [58] and can be released together with variable amounts of PGN by lysozyme treatment. For lipase induction, *S. carnosus* (pCX33TLCH) was grown to mid log phase in the presence of 0.5% xylose. Non-covalently bound proteins were removed by washing steps with and without sodium chloride at RT. Covalently PGN-bound proteins were excised by treatment with hen egg white lysozyme (0.2 mg/ml) at 37°C. Cell wall released proteins in the supernatant were denatured with 8 M urea to resolve protein clusters and for improved processing of the lipase propeptide by thrombin at the TCS. SHL-PGN was purified in a two-step process under denaturing conditions by ÄKTA-FPLC with a HisTrap column. Using lipase specific antibodies, SHL-PGN was analyzed by SDS-PAGE and Western blot.

### Purification of SHL from *S. carnosus* ΔRKET (pPSHG5ΩHis-SHL)

Mature lipase (without PP) with an N-terminal his-tag (SHL) was expressed intracellularly in *S. carnosus* ΔRKET (pPSHG5ΩHis-SHL) [59]. SHL was isolated under denaturing conditions with 8 M urea and subsequently purified by ÄKTA-FPLC with a HisTrap column.

### Purification of the lipoprotein SitC from *S. carnosus* (pTX30SitC-his)

The *sit*C gene was cloned into a derivative of the xylose-inducible expression vector pTX15 [60] and expressed with a C-terminal extension of twelve amino acids (thrombin cleavage-site and histidine tag; KVPRGSHHHHHH), named SitC-his. *sit*C was amplified from *S. aureus* SA113 genomic DNA using the primer pair *Bam*HI-fwd (TATTTAGGATCCGAAACGAGGAAGTTTAACATGAAAAAATTAG) and *Sac*I-rev (ATAATTGAGCTCTTAATGATGATGATGATGATGTGAACCACGTGGAACTAATTTCAG CTTCCGTGTAC) with the High Fidelity PCR enzyme mix (Fermentas, St.Leon-Rot). The sequence of the PCR product (999 bp) contained the *sit*C gene with a C-terminal thrombin-cleavable His_6_ tag. This PCR product was cloned in-frame into the polylinker upstream of the *xyl*A promoter region of the pTX15 derivative, resulting in pTX30sitC-His. This plasmid was transformed into *S. aureus* SA113 by electroporation [61] and the insert was sequenced using a LI-COR DNA sequencer Long Reader (Lincoln Corporation, Inc. Lincoln, Neb). Computer sequence analysis was performed using the Vector NTI program. Cells were grown in the presence of 0.5% xylose for 13h, harvested, and broken via FastPrep. Cell membranes were obtained by ultracentrifugation. pre-SitC contains an N-terminal leader sequence and a C-terminal histidine tag. The first step in the maturation of all lipoproteins is the transfer of the diacylglyceryl sulfhydryl group of the invariant cysteine residue in the leader sequence. This reaction is catalyzed by the phosphatidylglycerol-prelipoprotein diacylglyceryl transferase, encoded by the *lgt* gene [62]. A second enzyme, Lsp (lipotein signal peptidase), recognizes the diacylglyceryl modification and cleaves the leader sequence, when lipoproteins are translocated through the cytoplasmic membrane [63], resulting in the mature lipoprotein. Mature SitC-his was solubilized by Triton buffer for 18h at 6°C. SitC-his purification was performed by Ni-NTA (Qiagen, Hilden, Germany) with a two-step elution procedure. Eluates were combined and concentrated. The concentrated eluate was analyzed by SDS-Page. The total amount of purified SitC was estimated by Bradford assay and stored at −80°C.

### Determination of LPS contamination

LPS concentration was determined by QCL-1000 LAL assay (Cambrex, Walkersville, USA) (Table 2).

### Western blot

Blots were carried out as described in the QIA-Expressionist.

### Labeling of purified proteins

Proteins were resuspended in LPS-free 0.1M sodium carbonate buffer, pH 10–11. The Cy2- or Cy5-labeling occurred as recommended by the manufacturer (GE Healthcare, Freiburg, Germany). The samples were spun at 5000 g for 90 min at RT in a centrifugal ultrafiltration unit (cut-off 10 kDa) to remove unbound dye and change buffer conditions to neutral pH (LPS-free PBS). Fluorescence labeled proteins were finally diluted in 1 ml LPS-free PBS and stored at −80 C°.

### Reagents used in cell culture

Penicillin/streptomycin, accutase, non-essential amino acids, and PBS were obtained from PAA (Pasching, Austria). LPS from *E. coli* were purchased from Sigma-Aldrich (Taufkirchen, Germany). The synthetic lipopeptide N-palmitoyl-*S*-[ 2,3-bis(palmitoyloxy)-(2RS)-propyl]-(*R*) -cysteinyl -(lysyl) 3- lysine (Pam_3_Cys; EMC Microcollections, Tübingen, Germany), mirroring the triacylated lipoproteins of bacteria, is also known to affect TLR2 [64]. Antibodies used were rabbit anti-TLR2, rabbit anti-TLR4, rabbit anti-Nod2 (all Santa Cruz Biotechnology, Santa Cruz, USA), Cy3-conjugated donkey anti-rabbit (Jackson ImmunoResearch Lab., West Grove, USA) and FITC-conjugated goat anti-biotin (Sigma-Aldrich, Saint Loiuse, USA).

### Cells and cell lines

To obtain primary cultures of murine epithelial cells (keratinocytes), oral mucosa was prepared from sacrificed mice. After overnight treatment with epidermis upside-down in trypsin solution at 4°C, the epidermis was separated from the dermis, and epidermal cells were collected by centrifugation. Murine epithelial cells were cultured in defined medium (64,5% D-MEM, 21,5% Ham's, 10% fetal calf serum, 2% penicillin-streptomycin (10000 U/ml, 10 mg/ml) for 2 days at 37°C and 5% CO_2_. Subsequently, cells were transferred to a second medium (94% MCDB, 2% fetal calf serum, 2% penicillin-streptomycin) and grown for 3 days before starting experiments.

MonoMac 6 were cultured in RPMI 1640 medium (Biochrom AG, Berlin, Germany) supplemented with 10% fetal calf serum (Biochrom AG, Berlin, Germany), 2× non-essential amino acids and OPI-supplement (Sigma-Aldrich, Taufkirchen, Germany). Cells were grown for 3 days before starting experiments.

### Sample preparation for confocal microscopy

3×10^5^ freshly isolated, live keratinocytes were seeded in chamber slides for 3–5 days at 37°C, 5% CO_2_ and 95% relative humidity. Afterwards, cells were incubated with different stimulants for 15–180 min. Cells were fixed with PLP (0.1 M L-Lysin-HCL, 2% paraformaldehyde 0.01 M sodium-*meta*-periodate pH 7.4) for 10 min at RT and permeabilized with 0.5% Triton X-100, 10 min at RT. Cells were subsequently stained with antibodies against TLR2, Nod2 or TLR4 (all rabbit antibodies). A Cy3-conjugated anti-rabbit antibody was used as a secondary antibody. Biotin was detected by goat anti-biotin antibody. Nuclei were stained with DAPI (Invitrogen, Karlsruhe, Germany) or Yo-Pro-1 iodide (Invitrogen, Karlsruhe, Germany). Cells were observed by CLSM using a Leica TCS SP 2 spectral confocal and multiphoton inverted microscope (Leica, Heidelberg, Germany). Images were taken using a Plan-Apochromat 63×/1.32 oil immersion objective and fluorescence excitation at 488 nm (Ar-Laser), 543 nm (HeNe-Laser), 633 nm (HeNe-Laser) and a Enterprise II 351/364 nm DAPI-Laser (Coherent, Dieburg, Germany). Appropriate filters were selected for the individual staining. We used pinhole 1 to guarantee that only one focal plane with a thin section 0.4 µm was imaged. Fluorescence analysis was carried out by Leica confocal software.

### Cytokine ELISA

MonoMac 6 cells were stimulated with each 50 µg/ml or 100 µg/ml of PGN_pol_, NaIO_4_-oxidized PGN_pol_, as well as PGN-Bio, LPS and Pam_3_Cys for 4 or 24 h at 37°C in a humidified 5% CO_2_ environment. MK were seeded in 500 µl/well in 24 well plates at a density of 10^5^ cells/ml. Cells were cultured in defined medium for 2 days at 37°C and 5% CO_2_. Subsequently, cells were transferred to a second medium and grown for 3 days before starting experiments. MK were stimulated with different amounts of PGN-Bio as well as with LPS for 48 h. The cell supernatants were collected and stored at −20°C. Human IL-6, human TNF-α and murine IL-6 levels were determined by ELISA kits according to the manufacturers' instructions (eBioscience, San Diego, USA). The optical density was measured at 450 nm and 570 nm by the Tecan Infinite 200 Reader (Tecan, Crailsheim, Germany).

### Dot blot analysis and detection of biotinylated PGN

PGN_pol_ suspended in deionized water was applied to a nitrocellulose membrane. The membrane was dried in air for 3 h and blocked with 0.5% casein (Merck, Darmstadt, Germany) in TTBS for 1 h at RT. The membrane was washed three times with TTBS. Horseradish peroxidase-conjugated streptavidin (streptavidin-HRP) was applied in TTBS with 0.5% casein for 1.5 h at RT. Four washing steps with TTBS were followed by visualization of the biotin via enhanced chemoluminescence (ECL-kit from Amersham Biosciences, Uppsala, Sweden).

### Reporter assay for NFkB activation

Human embryonic kidney (HEK293) cells (ATCC, Manassas, VA) were cultured in supplemented DMEM (2mM L-Glutamine and 10% fetal calf serum; all from Biochrom, Berlin, Germany). HEK293, stable transfected HEK293-hTLR2 cells (Invivogen, San Diego, CA, USA) and freshly transfected HEK293-hNod2 cells (pUNO-hNOD2a; InvivoGen, San Diego USA) were plated at 2×10^5^ cells/well in 24-well plates one day before transfection. Cells were then transiently transfected by Lipofectamine 2000 transfection reagent (InvivoGen, San Diego USA) with 100 ng of a NFkB-reporter plasmid (pNFkB-TA-Luc, Clontech, BD Biosciences, Heidelberg, Germany) and 10 ng of a plasmid that directed expression of renilla luciferase under the control of the constitutively active thymidine kinase promoter (pRL-TK, Promega). The pcDNA3.1 vector (Promega) was used to balance the transfected DNA concentration. Twenty-four hours after transfection cells were stimulated with cell wall components in serum free medium for 24 h and luciferase activity was measured using the dual luciferase reporter assay system (Promega) according to manufacturer's instructions.

### Ethics statement

C57BL/6 wild-type mice were purchased from Charles River (Sulzfeld, Germany) and Nod2-deficient mice from Jackson Laboratory (Maine, USA). TLR2-deficient mice were obtained from C. Kirschning (Technical University, Munich). All deficient strains were in a C57BL/6 background and bred under specific pathogen-free conditions at the animal facility of the University of Tübingen according to European guidelines and German law. Preparation of oral mucosa has been approved by the Regierungspräsidium Tübingen (Az Anzeige vom 25.04.07 and Anzeige vom 05.06.09).

## Supporting Information

Figure S1 HPLC profile of mutanolysin digested PGN_pol_. Muropeptides were detected at 205 nm.
(3.62 MB TIF)

Figure S2 Incorporation of PGN-Bio in MK and co-localization with Nod2 and TLR2 is time- and concentration dependent. Confocal images of MK stained intracellularly with a Nod2-antibody (A–C) or a TLR2-antibody (D–F). PGN-Bio from was detected by a FITC-conjugated anti-biotin-antibody (green). The upper panels show the merging images; co-localization events are visualized in yellow. The lower images show an overlay of fluorescence merge and the host cell acquired in reflection mode of the confocal microscope. Wt MK were stimulated with different amounts of PGN-Bio for various time periods. Images of cells shown are representative of the cells observed in each dish and are representative of three experiments.
(9.94 MB TIF)

Figure S3 PGN_pol_ did not affect TLR4. Confocal images of MK stained with a TLR4-antibody from rabbit (detected by a Cy3-conjugated anti-rabbit antibody [red]). Nuclei were stained with DAPI (blue). PGN-Bio was detected by a FITC-conjugated anti-biotin antibody (green). The upper panels show the merging images. The lower images show an overlay of fluorescence merge and the host cell acquired in reflection mode of the confocal microscope at 488 nm. (A) PBS control. (B) No TLR4 was detected after stimulation with PGN-Bio. (C) TLR4 was detected after stimulation with LPS in MK. Images of cells shown are representative of the cells observed in each dish and are representative of three experiments.
(5.74 MB TIF)

Figure S4 Nod2 and TLR2-dependent NFκB activation mediated by PGN_pol_. Reporter assay with NFκB-reporter plasmid (pNFκB-TA-Luc) transfected HEK293 cells. Without any PRR (1), hTLR2 expressing HEK293 (2) and hNod2 expressing HEK293(3). Cells were stimulated with different amounts of PGN_pol_. PGN_pol_ showed a both Nod2 and TLR2-dependent activity. The data were shown as the mean ± S.D. from three independent experiments.
(1.72 MB TIF)
