# Supplementary figures and images for: Staphylococcal Peptidoglycan Co-Localizes with Nod2 and TLR2 and Activates Innate Immune Response via Both Receptors in Primary Murine Keratinocytes
Source: PLoS One. 2010 Oct 7;5(10):e13153. doi: 10.1371/journal.pone.0013153 (PMC2951902; doi:10.1371/journal.pone.0013153)

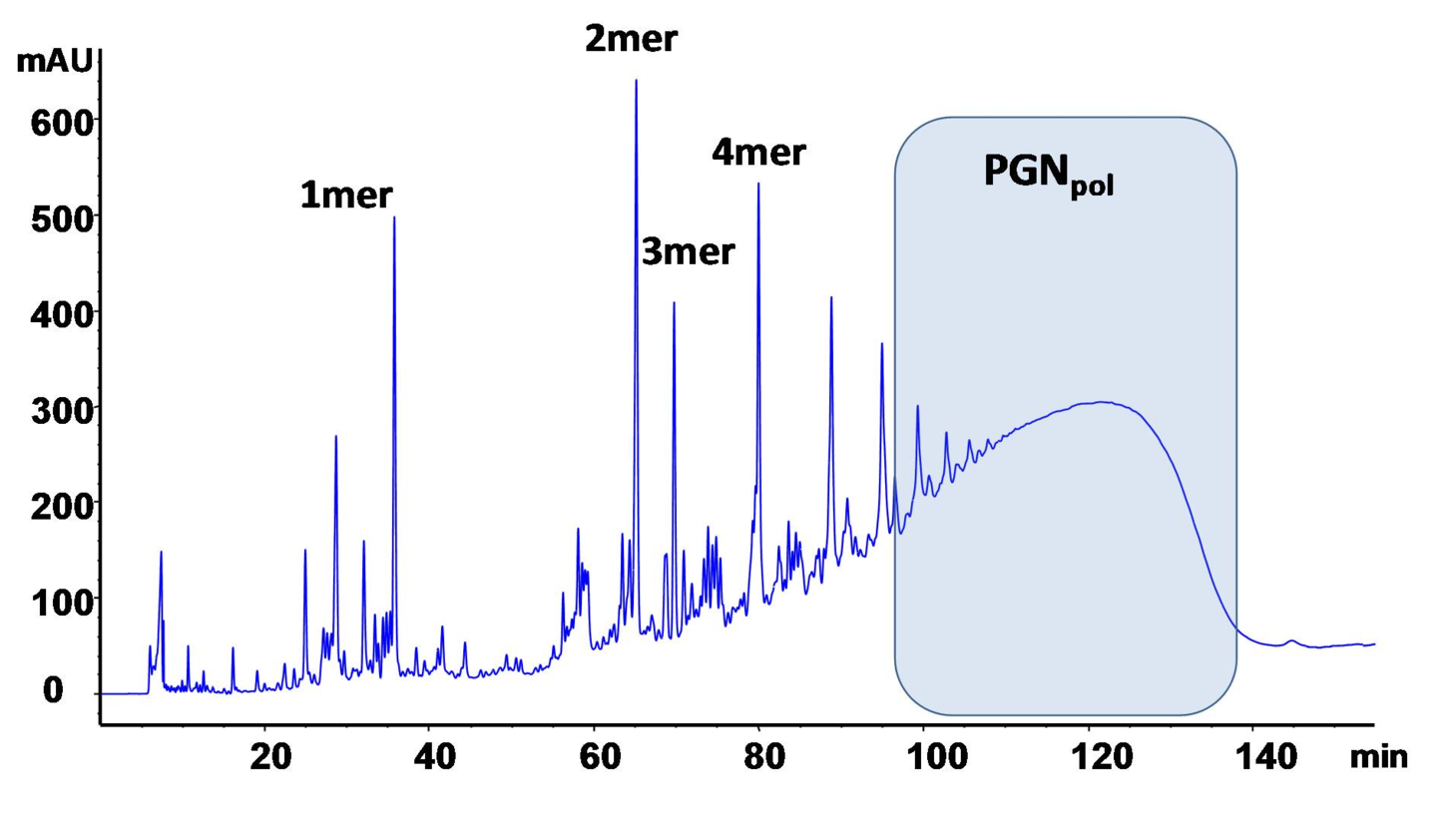

Supplement: Figure S1 — HPLC profile of mutanolysin digested PGNpol. Muropeptides were detected at 205 nm. (3.62 MB TIF) [file pone.0013153.s001.tif]

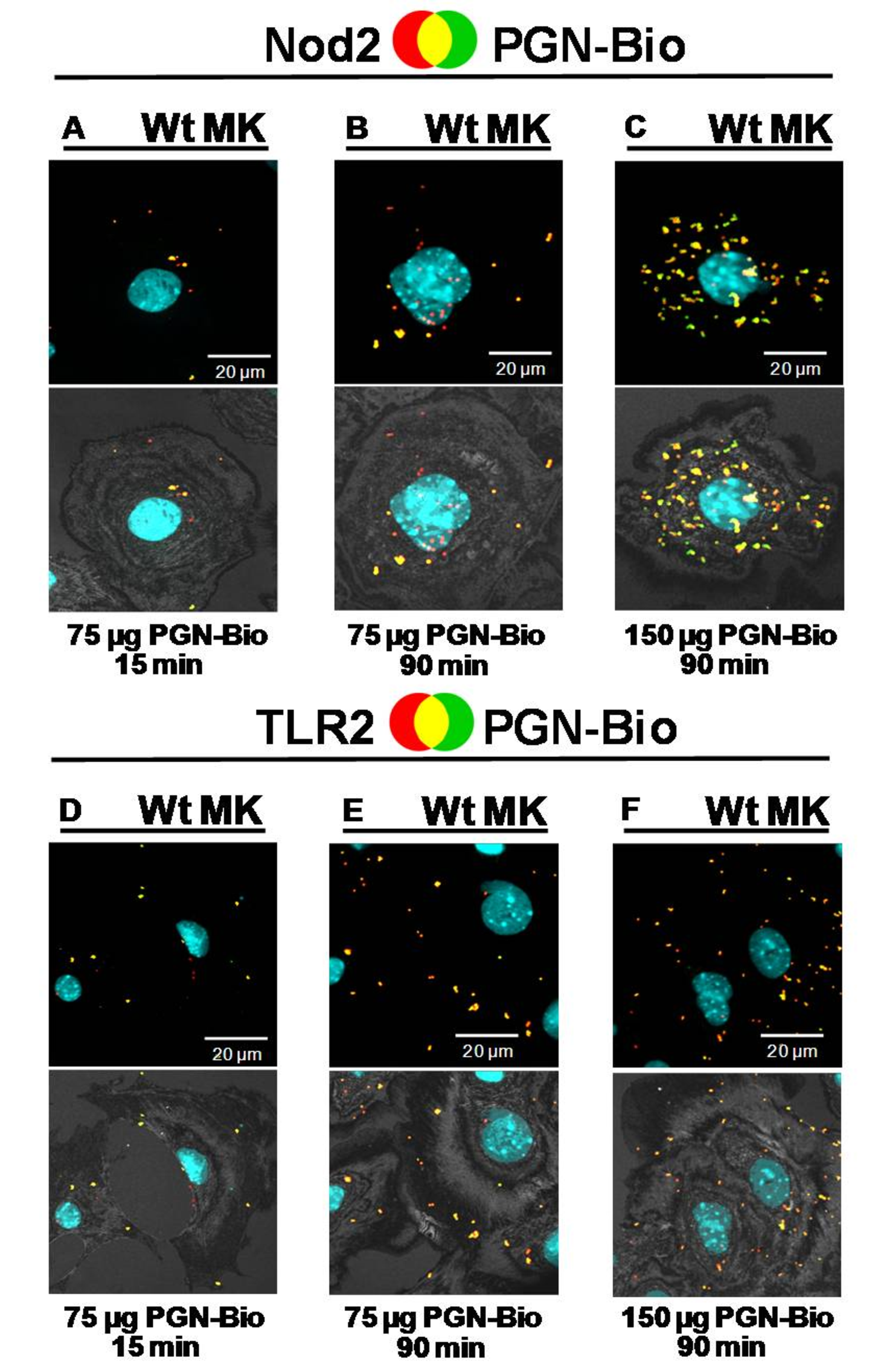

Supplement: Figure S2 — Incorporation of PGN-Bio in MK and co-localization with Nod2 and TLR2 is time- and concentration dependent. Confocal images of MK stained intracellularly with a Nod2-antibody (A–C) or a TLR2-antibody (D–F). PGN-Bio from was detected by a FITC-conjugated anti-biotin-antibody (green). The upper panels show the merging images; co-localization events are visualized in yellow. The lower images show an overlay of fluorescence merge and the host cell acquired in reflection mode of the confocal microscope. Wt MK were stimulated with different amounts of PGN-Bio for various time periods. Images of cells shown are representative of the cells observed in each dish and are representative of three experiments. (9.94 MB TIF) [file pone.0013153.s002.tif]

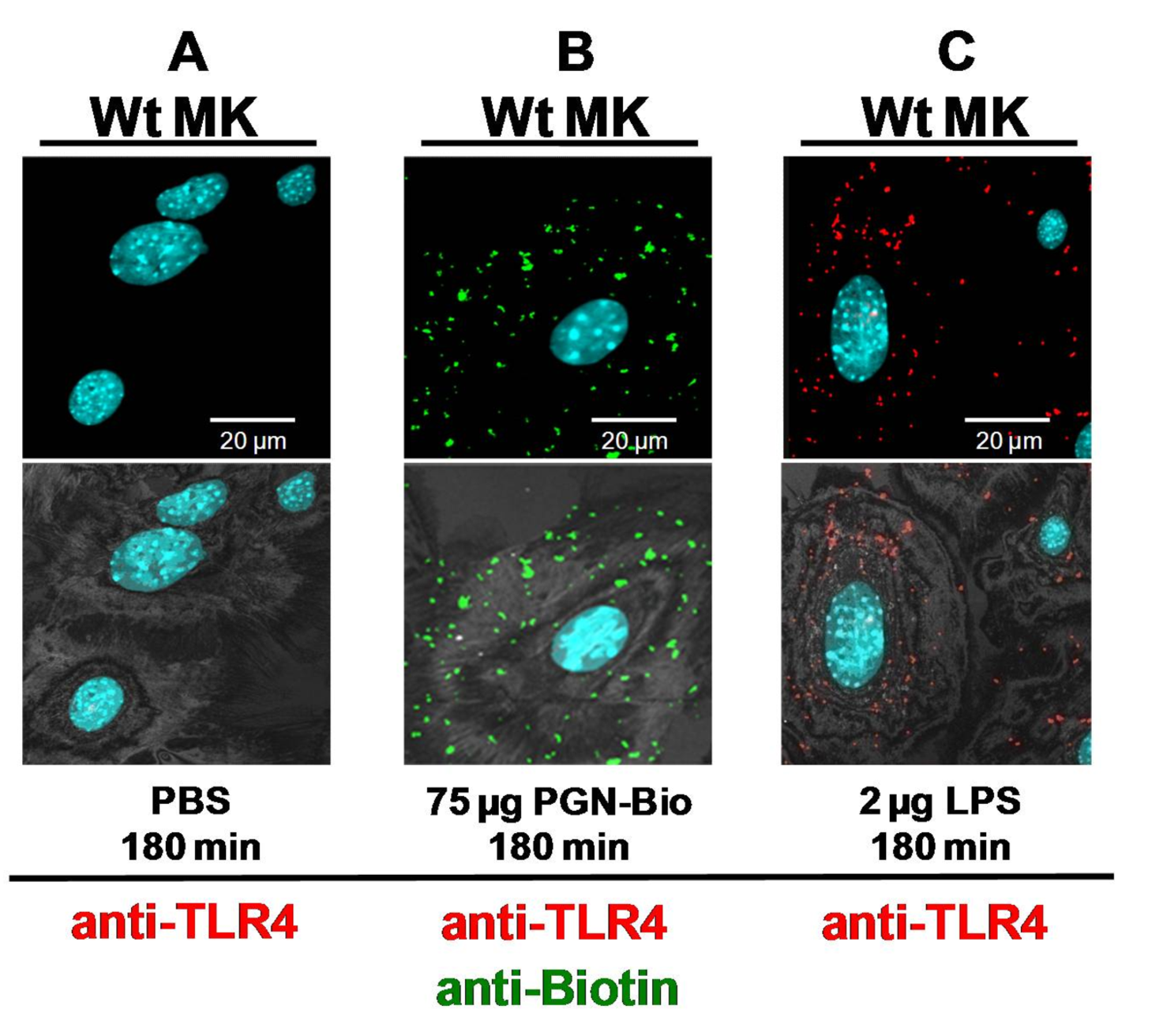

Supplement: Figure S3 — PGNpol did not affect TLR4. Confocal images of MK stained with a TLR4-antibody from rabbit (detected by a Cy3-conjugated anti-rabbit antibody [red]). Nuclei were stained with DAPI (blue). PGN-Bio was detected by a FITC-conjugated anti-biotin antibody (green). The upper panels show the merging images. The lower images show an overlay of fluorescence merge and the host cell acquired in reflection mode of the confocal microscope at 488 nm. (A) PBS control. (B) No TLR4 was detected after stimulation with PGN-Bio. (C) TLR4 was detected after stimulation with LPS in MK. Images of cells shown are representative of the cells observed in each dish and are representative of three experiments. (5.74 MB TIF) [file pone.0013153.s003.tif]

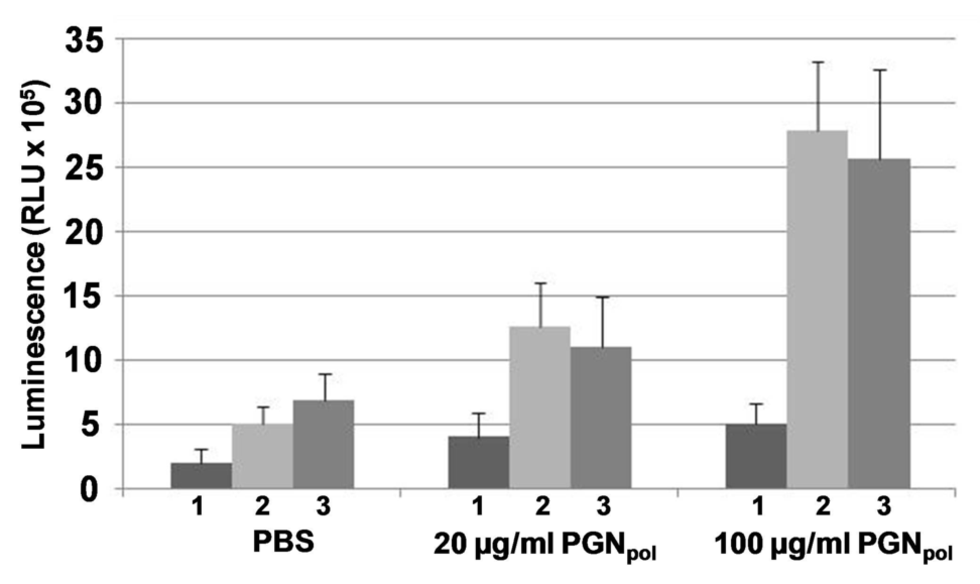

Supplement: Figure S4 — Nod2 and TLR2-dependent NFκB activation mediated by PGNpol. Reporter assay with NFκB-reporter plasmid (pNFκB-TA-Luc) transfected HEK293 cells. Without any PRR (1), hTLR2 expressing HEK293 (2) and hNod2 expressing HEK293(3). Cells were stimulated with different amounts of PGNpol. PGNpol showed a both Nod2 and TLR2-dependent activity. The data were shown as the mean ± S.D. from three independent experiments. (1.72 MB TIF) [file pone.0013153.s004.tif]
